# Global evaluation of echocardiography in patients with COVID-19

**DOI:** 10.1093/ehjci/jeaa178

**Published:** 2020-06-18

**Authors:** Marc R Dweck, Anda Bularga, Rebecca T Hahn, Rong Bing, Kuan Ken Lee, Andrew R Chapman, Audrey White, Giovanni Di Salvo, Leyla Elif Sade, Keith Pearce, David E Newby, Bogdan A Popescu, Erwan Donal, Bernard Cosyns, Thor Edvardsen, Nicholas L Mills, Kristina Haugaa

**Affiliations:** j1 Centre for Cardiovascular Science, University of Edinburgh, UK; j2 Columbia University Irving Medical Center, NY, USA; j3 University Hospital Padua, Paediatric Cardiology, Padua, Italy; j4 Department of Cardiology, University of Baskent, Ankara, Turkey; j5 University Hospital South Manchester, Cardiology, Wythenshawe, Manchester, UK; j6 Department of Cardiology, University of Medicine and Pharmacy ‘Carol Davila’-Euroecolab, Emergency Institute for Cardiovascular Diseases ‘Prof. Dr. C. C. Iliescu’, Bucharest, Romania; j7 University of Rennes, CHU Rennes, Inserm, LTSI-UMR 1099, Rennes, France; j8 Centrum voor Hart en Vaatziekten, Universitair Ziekenhuis Brussel, Vrij Universiteit van Brussel, Brussels, Belgium; j9 Department of Cardiology, Oslo University Hospital, Rikshospitalet, Oslo, Norway; j10 Faculty of Medicine, University of Oslo, Oslo, Norway; j11 Usher Institute, University of Edinburgh, UK

**Keywords:** COVID-19, Echocardiography

## Abstract

**Aims:**

To describe the cardiac abnormalities in patients with COVID-19 and identify the characteristics of patients who would benefit most from echocardiography.

**Methods and results:**

In a prospective international survey, we captured echocardiography findings in patients with presumed or confirmed COVID-19 between 3 and 20 April 2020. Patient characteristics, indications, findings, and impact of echocardiography on management were recorded. Multivariable logistic regression identified predictors of echocardiographic abnormalities. A total of 1216 patients [62 (52–71) years, 70% male] from 69 countries across six continents were included. Overall, 667 (55%) patients had an abnormal echocardiogram. Left and right ventricular abnormalities were reported in 479 (39%) and 397 (33%) patients, respectively, with evidence of new myocardial infarction in 36 (3%), myocarditis in 35 (3%), and takotsubo cardiomyopathy in 19 (2%). Severe cardiac disease (severe ventricular dysfunction or tamponade) was observed in 182 (15%) patients. In those without pre-existing cardiac disease (*n* = 901), the echocardiogram was abnormal in 46%, and 13% had severe disease. Independent predictors of left and right ventricular abnormalities were distinct, including elevated natriuretic peptides [adjusted odds ratio (OR) 2.96, 95% confidence interval (CI) 1.75–5.05) and cardiac troponin (OR 1.69, 95% CI 1.13–2.53) for the former, and severity of COVID-19 symptoms (OR 3.19, 95% CI 1.73–6.10) for the latter. Echocardiography changed management in 33% of patients.

**Conclusion:**

In this global survey, cardiac abnormalities were observed in half of all COVID-19 patients undergoing echocardiography. Abnormalities were often unheralded or severe, and imaging changed management in one-third of patients.

## Introduction

Coronavirus disease 2019 (COVID-19) has emerged as a major cause of morbidity and mortality that is placing unprecedented pressure on healthcare services across the world.^1^^,^^2^ Whilst the severe acute respiratory syndrome coronavirus 2 (SARS-CoV-2) responsible for COVID-19 predominantly affects the respiratory tract,^3^ patients with cardiovascular risk factors or established disease^4^ and those with elevated cardiac biomarkers appear to be more susceptible and to have a worse prognosis.^5^^,^^6^ The mechanisms underlying these initial observations remain unclear.

Early case reports suggest that COVID-19 can cause a wide range of cardiac conditions that include acute myocardial infarction,^7^ myocarditis,^8^ and takotsubo cardiomyopathy.^9^ Acute left and right ventricular failure may be a direct consequence of cardiac pathology, with the latter also arising secondary to elevations in right ventricular afterload due to pulmonary embolism or pneumonia.^10^ Virus particles have been observed in the myocardium and vascular endothelium in patients with COVID-19 and cardiogenic shock.^11^^,^^12^ However, the incidence of these cardiac complications and the subsequent implications for treatment and resource allocation are unknown. Consequently, there is an urgent need to better understand the interactions between COVID-19 and the heart.

Echocardiography is well placed to help further this understanding, being inexpensive, portable, and widely accessible. However, a large systematic evaluation of echocardiography in all patients with COVID-19 would be highly challenging due to the logistical considerations of testing, consumption of personal protective equipment (PPE), and the risk of further viral transmission. We therefore conducted a global survey to capture the findings of echocardiography performed on clinical grounds in patients with confirmed or a high probability of COVID-19. We aim to improve our understanding of the cardiac manifestations of COVID-19, and to provide insights into the characteristics of patients who would benefit most from echocardiography.

## Methods

This global online survey of echocardiography in patients with COVID-19 was designed by the European Association of Cardiovascular Imaging (EACVI), with input from external international experts.^13^ Any transthoracic echocardiogram that was performed on a patient with confirmed, or a high probability of, COVID-19 in the hospital setting was eligible for inclusion. Patients were imaged as part of routine care, and non-identifiable patient data were captured. As such, this audit did not require individual patient consent and this approach was approved by the European Society of Cardiology and by local research ethics committees.

An online format was developed (https://www.surveymonkey.com/r/2FBFFQD) that allows rapid completion of 11 questions on a smartphone by sonographers or clinicians immediately after completion of the echocardiogram. For most questions, the operator selects from several pre-specified answers, with the option to select multiple answers and to provide free-text comments (Supplementary material online, *Appendix*). First, several baseline characteristics are recorded: age, sex, comorbidities, symptom severity, COVID-19 status, presence of pneumonia, and the location in hospital where imaging was performed. Secondly, the indication for imaging is recorded: suspected left heart failure, suspected right heart failure, chest pain with ST-segment elevation on the electrocardiogram, cardiac biomarker elevation [troponin or brain natriuretic peptide (BNP)], ventricular arrhythmia, suspected tamponade, or cardiogenic shock. Thirdly, echocardiographic findings are captured for left ventricular abnormalities (normal, mild, moderate, or severe systolic dysfunction, dilatation, evidence of new myocardial infarction, myocarditis, or takotsubo cardiomyopathy), right ventricular abnormalities (normal, mild or moderate, or severe systolic dysfunction, dilatation, D-shaped left ventricle, or elevated pulmonary artery pressure), or cardiac tamponade. The survey then captures whether the echocardiogram changed patient management.

This prospective survey (www.escardio.org/eacvi/surveys) was distributed to the EACVI network and its wider membership,^14^^,^^15^ to a pre-established European Society of Cardiology database of cardiologists with an interest in cardiac imaging, and to the presidents and chairpersons of national societies and working groups in imaging across the world. It was also distributed widely on social media platforms.

## Statistical analysis

Survey entries were excluded if there were incompatible, incomplete, or conflicting data, or if there were no echocardiographic findings recorded. In this analysis, missing values were not imputed. Age was reported as median with an interquartile interval, and categorical variables were reported as frequencies (%). Between-group comparisons were performed using the χ^2^ test or an independent samples *t*-test. In the primary analysis, patients with either confirmed or probable COVID-19 were included. A sensitivity analysis was performed restricted to those with confirmed COVID-19. A critical care setting was defined as intensive care, high dependency, or coronary care units, the emergency department, or the cardiac catheterization laboratory. A normal echocardiogram was defined as normal left and right ventricular function, with no other reported abnormalities. To evaluate associations between clinical variables and cardiac abnormalities that were more likely to be due to COVID-19, an analysis was performed in patients without pre-existing cardiac disease, after excluding those with previous ischaemic heart disease, heart failure, or valvular heart disease. Univariable and multivariable logistic regression models were constructed separately with an abnormal left ventricle (any degree of left ventricular dysfunction or dilatation, myocardial infarction, myocarditis, or takotsubo cardiomyopathy) or abnormal right ventricle (any right ventricular dysfunction or dilatation, a D-shaped left ventricle, or pulmonary hypertension) as the dependent variables. Covariates included age, gender, scan location, symptom severity, hypertension and diabetes mellitus, and the indication for echocardiography. Analysis was performed using R version 3.5.0 (R Foundation for Statistical Computing, Vienna, Austria).

## Results

**Figure 1:**
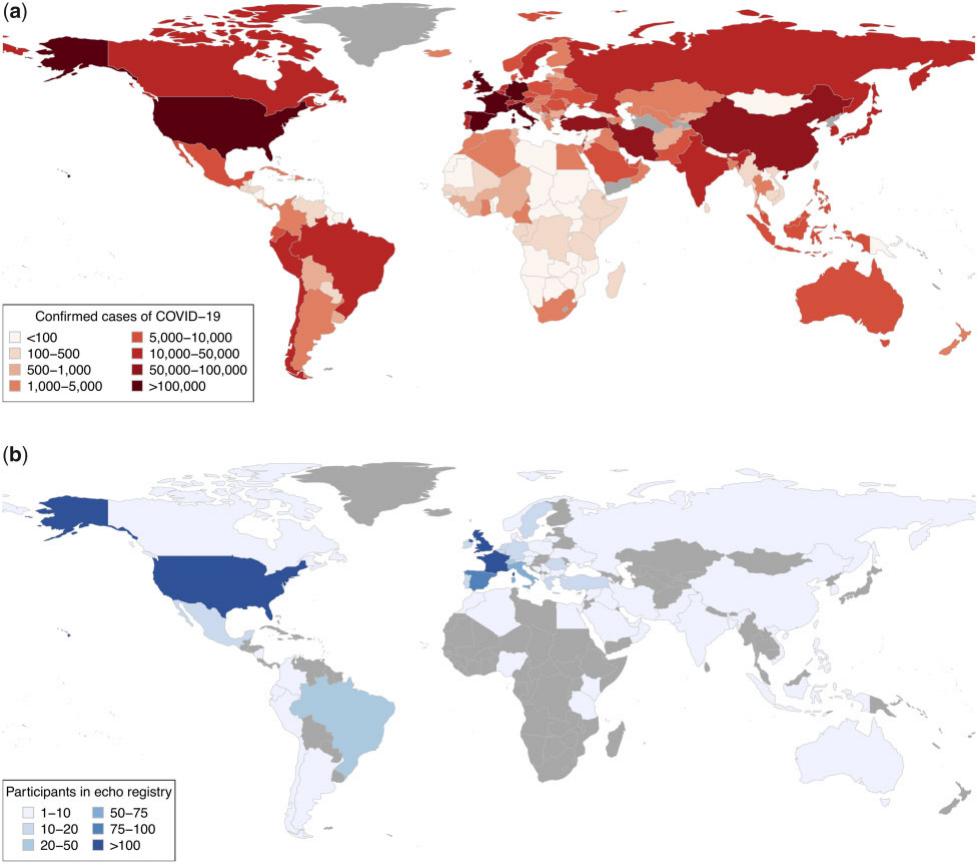
Prevalence of COVID-19 and countries contributing to the global online survey of echocardiography. (*A*) The prevalence of COVID-19 on 20 April for the 185 countries for which data were available through the Johns Hopkins Center for Systems Science and Engineering COVID-19 dashboard.^25^ (*B*) The location and number of scans reported in the global online survey of echocardiography during a 17-day period from 3 to 20 April 2020.

The survey was launched on 3 April 2020. The results reported here comprise data from the first 17 days of the survey, with the last date of collection on 20 April 2020. Data from 1272 patients undergoing echocardiography were collected from 69 countries across six continents where COVID-19 has been reported (*Figure 1*). Data were available for analysis in 1216 (96%) patients [62 (52–71) years, 70% male], of whom 813 (73%) had confirmed COVID-19, and 298 (27%) had a high probability at the time of scanning (*Table 1*).

**Table 1:** Patient characteristics and indications for echocardiography

	Overall (*n* = 1216)	Abnormal scan (*n* = 667)	Normal scan (*n* = 549)	*P*-value*
Age	62 (52–71)	64 (53–73)	60 (51–69)	<0.001
Sex				0.600
Female	365 (30%)	195 (29%)	170 (31%)	
Male	844 (70%)	468 (71%)	376 (69%)	
Location of scan				0.053
Critical care	726 (60%)	382 (57%)	344 (63%)	
Non-critical care	486 (40%)	284 (43%)	202 (37%)	
COVID-19 status				<0.001
Confirmed	813 (73%)	409 (68%)	404 (79%)	
High probability	298 (27%)	193 (32%)	105 (21%)	
Evidence of pneumonia	232 (19%)	135 (20%)	97 (18%)	0.300
Symptom severity				<0.001
Mild	215 (18%)	98 (15%)	117 (23%)	
Moderate	327 (28%)	210 (32%)	117 (23%)	
Severe	625 (54%)	340 (52%)	285 (55%)	
Co-morbidities				
Hypertension	445 (37%)	254 (38%)	191 (35%)	0.300
Diabetes mellitus	233 (19%)	136 (20%)	97 (18%)	0.300
Ischaemic heart disease	167 (14%)	137 (21%)	30 (6%)	<0.001
Heart failure	113 (9%)	106 (16%)	7 (1%)	<0.001
Valvular heart disease	80 (7%)	53 (8%)	27 (5%)	0.045
Indication				
Suspected left heart failure	491 (40%)	294 (44%)	197 (36%)	0.011
Suspected right heart failure	243 (20%)	145 (22%)	98 (18%)	0.200
Chest pain and ST-elevation	107 (9%)	76 (11%)	31 (6%)	0.001
Elevated cardiac biomarkers	314 (26%)	216 (32%)	98 (18%)	<0.001
Troponin	239 (20%)	164 (25%)	75 (14%)	<0.001
BNP	129 (11%)	97 (15%)	32 (6%)	<0.001
Ventricular arrhythmia	38 (3%)	33 (5%)	5 (1%)	<0.001
Cardiac tamponade	20 (2%)	13 (2%)	7 (1%)	0.600
Circulatory shock	95 (8%)	65 (20%)	30 (6%)	0.017
Change in management				<0.001
Yes	405 (33%)	297 (45%)	108 (20%)	
No	675 (56%)	309 (46%)	366 (67%)	
Not known	136 (11%)	61 (9%)	75 (14%)	
Management group				<0.001
Disease-specific therapy	171 (14%)	130 (19%)	41 (8%)	
Level of care	32 (3%)	20 (3%)	12 (2%)	
Haemodynamic support	51 (4%)	35 (5%)	16 (3%)	
Other	151 (12%)	112 (17%)	39 (7%)	

Median (interquartile range), number (%). Abbreviations: BNP, brain B-type natriuretic peptide; COVID-19, coronavirus disease 2019.

* Between-group comparisons are χ^2^ test or independent samples *t*-tests

Missing values in the overall population: age = 18; sex = 7; location of scan = 4; COVID-19 status = 8; symptom severity = 49; indication = 9.

Overall, 60% of scans were performed in a critical care setting (54% intensive care, 2% high dependency unit and coronary care unit, 5% emergency room, and 1% cardiac catheter laboratory), with the remainder performed in general medicine, cardiology, respiratory, and dedicated COVID-19 wards (*Table 1*). Correspondingly, 54% of patients had severe symptoms and 19% had evidence of pneumonia. Pre-existing cardiac disease was reported in 26% of patients due to a combination of ischaemic heart disease (14%), heart failure (9%), or valvular heart disease (7%). Hypertension (37%) and diabetes mellitus (19%) were also common. The most common indications for echocardiography were suspected left-sided heart failure (40%), elevated cardiac biomarkers (26%), and right-sided heart failure (20%). Chest pain with ST-segment elevation on the electrocardiogram (9%), circulatory shock (8%), ventricular arrhythmia (3%), and suspected cardiac tamponade (2%) were less frequent, as were other indications, such as suspected pulmonary embolism (5%), endocarditis (6%), and myocarditis (1%).

## Echocardiographic findings

**Figure 2:**
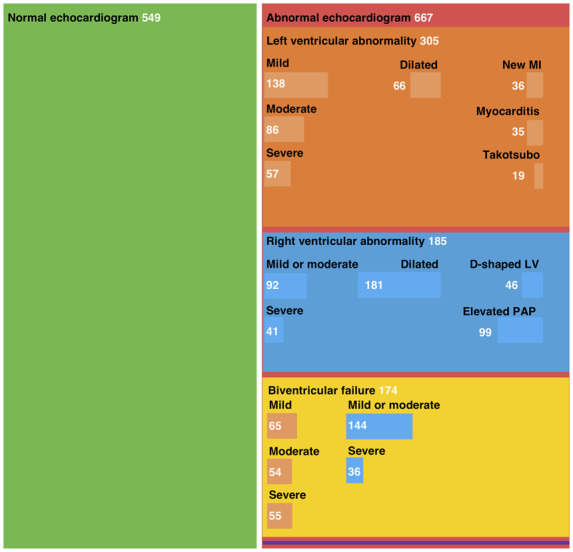
Central Illustration. Mosaic plot illustrating the findings on echocardiography in patients with COVID-19. Mosaic plot illustrating the distribution of normal and abnormal echocardiogram findings in patients with suspected or confirmed COVID-19 infection. Box size is proportional to the number of patients per category. Left ventricular abnormalities are shown in orange and right ventricular abnormalities are shown in blue, both in the independent boxes and in the biventricular failure box. Survey respondents could enter data for multiple categories of left or right ventricular abnormality, therefore subcategories are not mutually exclusive. Eleven patients had evidence of cardiac tamponade which was an isolated finding in three patients, illustrated in purple. LV, left ventricle; MI, myocardial infarction; PAP, pulmonary arterial pressure.

Compared with patients with a normal echocardiogram (*n* = 549, 45%), patients with an abnormal scan (*n* = 667, 55%) were older and had a higher prevalence of pre-existing ischaemic heart disease, heart failure, or valvular heart disease, but a similar prevalence of hypertension or diabetes mellitus. The proportion of males was similar in both groups (*Table 1*; *Figure 2*, *Central Illustration*).

Left ventricular abnormalities were reported in 479 (39%) patients, with echocardiographic evidence of new myocardial infarction in 36 (3%), myocarditis in 35 (3%), and takotsubo cardiomyopathy in 19 (2%). Left ventricular impairment was classified as mild, moderate, or severe in 17, 12, and 9% of patients, respectively. Right ventricular abnormalities were reported in 397 (33%) patients, with mild or moderate right ventricular impairment in 19% and severe impairment in 6%. Right ventricular dilatation (15%), elevated pulmonary artery pressures (8%), and a D-shaped left ventricle (4%) were reported less frequently. Cardiac tamponade and endocarditis were reported in 11 (1%) and 14 (1%) patients, respectively. Severe cardiac disease, defined as severe left or right ventricular dysfunction or cardiac tamponade, was reported in 1 in 7 patients (*n* = 182, 15%; Supplementary material online, *Table S1*).

**Figure 3:**
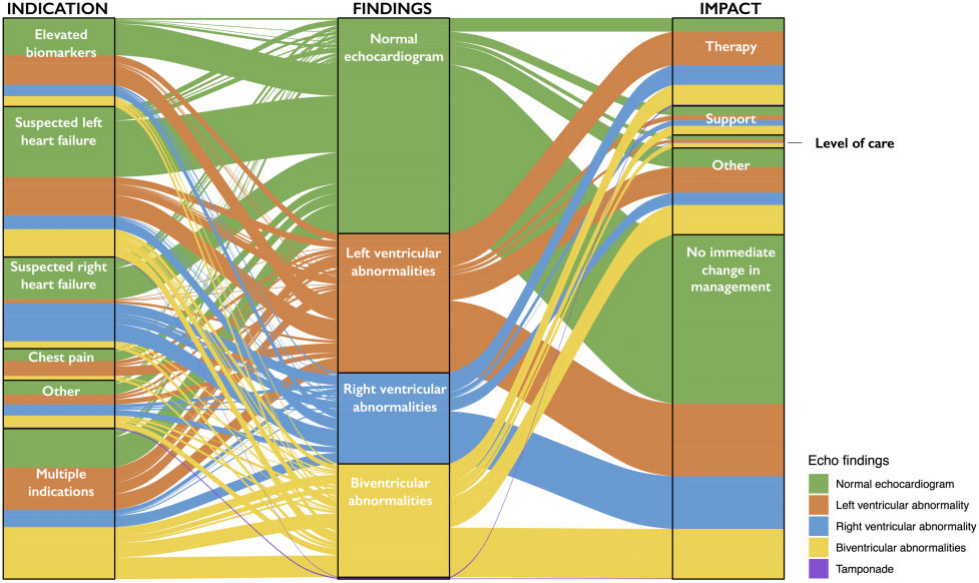
Alluvial plot illustrating the indications for echocardiography and impact on patient management grouped according to the scan findings. Colours represent findings reported on echocardiography. All patients for whom complete data were available for indication, findings, and change in management were included in this plot [89% (1080/1216)]. The elevated biomarker indication subgroup includes both indication for raised troponin and indication for elevated BNP. Changes in management (impact) include those where changes in disease-specific therapy, titration of haemodynamic support, or changes in the level of patient care were described.

Abnormalities on the echocardiogram were more common in those where the indication for imaging was chest pain with ST-segment elevation (71%), elevated biomarkers (69%), suspected left ventricular failure (60%), suspected right ventricular failure (60%), or where multiple indications were present (72%) (*Table 2*; *Figure 3*). In a sensitivity analysis restricted to the 813 patients with confirmed COVID-19, the proportion with an abnormal echocardiogram was similar to the overall population at 50% (409/813), and 1 in 7 patients had severe cardiac disease (*n* = 119, 15%).

**Table 2:** Echocardiographic findings stratified by indication

	Overall*^†^ (*n* = 1216)	Suspected left heart failure (*n* = 491)	Suspected right heart failure (*n* = 243)	Chest pain and ST elevation (*n* = 107)	Elevated cardiac biomarkers (*n* = 314)	Elevated troponin (*n* = 239)	Elevated BNP (*n* = 129)	Multiple indications (*n* = 276)	Other^‡^ (*n* = 299)
Overall findings									
Normal echocardiogram	549 (44%)	197 (40%)	98 (40%)	31 (29%)	98 (31%)	75 (31%)	32 (25%)	76 (28%)	180 (60%)
Abnormal echocardiogram	667 (53%)	294 (60%)	145 (60%)	76 (71%)	216 (69%)	164 (69%)	97 (75%)	200 (72%)	119 (40%)
Severe cardiac disease^§^	182 (15%)	81 (16%)	40 (16%)	11 (10%)	62 (20%)	44 (18%)	33 (26%)	63 (23%)	40 (13%)
Left ventricle*									
Normal	745 (61%)	247 (50%)	186 (77%)	33 (31%)	139 (44%)	109 (46%)	45 (35%)	114 (41%)	223 (75%)
Mild impairment	203 (17%)	92 (19%)	33 (14%)	38 (36%)	74 (24%)	60 (25%)	32 (25%)	66 (24%)	33 (11%)
Moderate impairment	140 (12%)	81 (16%)	10 (4%)	22 (21%)	50 (16%)	32 (13%)	25 (19%)	41 (15%)	18 (6%)
Severe impairment	112 (9%)	66 (13%)	12 (5%)	9 (8%)	45 (14%)	32 (13%)	26 (20%)	49 (18%)	21 (7%)
Dilated	66 (5%)	40 (8%)	8 (3%)	7 (7%)	31 (10%)	22 (9%)	19 (15%)	31 (11%)	11 (4%)
Evidence of new MI	36 (3%)	13 (3%)	4 (2%)	14 (13%)	22 (7%)	22 (9%)	7 (5%)	19 (7%)	4 (1%)
Evidence of myocarditis	35 (3%)	21 (4%)	4 (2%)	8 (8%)	24 (8%)	19 (8%)	13 (10%)	24 (9%)	4 (1%)
Evidence of takotsubo	19 (2%)	5 (1%)	1 (1%)	4 (4%)	11 (4%)	10 (4%)	5 (4%)	8 (3%)	6 (2%)
Right ventricle*									
Normal	842 (69%)	335 (68%)	124 (51%)	79 (74%)	206 (66%)	158 (66%)	79 (61%)	163 (59%)	224 (75%)
Mild to moderate impairment	236 (19%)	100 (20%)	64 (26%)	23 (21%)	79 (25%)	61 (26%)	37 (29%)	79 (29%)	48 (16%)
Severe impairment	77 (6%)	27 (6%)	32 (13%)	4 (4%)	20 (6%)	14 (6%)	9 (7.0%)	22 (8%)	16 (5%)
Dilated	181 (15%)	56 (11%)	76 (31%)	5 (5%)	44 (14%)	33 (14%)	21 (16%)	48 (17%)	41 (14%)
D-shaped left ventricle	46 (4%)	10 (2%)	22 (9%)	0 (0%)	8 (3%)	5 (2%)	6 (5%)	8 (3%)	12 (4%)
Elevated PAP	99 (8%)	31 (6%)	46 (19%)	3 (3%)	33 (11%)	23 (10%)	15 (12%)	31 (11%)	18(6%)
Other									
Tamponade	11 (1%)	3 (1%)	1 (1%)	0 (0%)	2 (1%)	2 (1%)	1 (1%)	3 (1%)	6 (2%)
Endocarditis	14 (1%)	3 (1%)	1 (1%)	0 (0%)	2 (1%)	1 (1%)	1 (1%)	2 (1%)	11 (4%)
Change in management									
Yes	405 (33%)	169 (34%)	85 (35%)	41 (38%)	123 (39%)	96 (40%)	53 (41%)	119 (43%)	96 (32%)
No	675 (56%)	243 (49%)	118 (49%)	62 (58%)	178 (57%)	133 (56%)	73 (57%)	134 (49%)	182 (61%)
Not known	136 (11%)	79 (16%)	40 (16%)	4 (4%)	13 (4%)	10 (4%)	3 (2%)	23 (8%)	21 (7%)
Management group									
Disease-specific therapy	171 (14%)	63 (13%)	38 (16%)	16 (15%)	53 (17%)	39 (16%)	26 (20%)	47 (17%)	42 (14%)
Level of care	32 (3%)	9 (2%)	3 (1%)	3 (3%)	6 (2%)	6 (3%)	1 (1%)	4 (1%)	13 (4%)
Haemodynamic support	51 (4%)	21 (4%)	11 (5%)	4 (4%)	12 (4%)	10 (4%)	3 (2%)	16 (6%)	14 (5%)
Other	151 (12%)	76 (15%)	33 (14%)	18 (17%)	52 (17%)	41 (17%)	23 (18%)	52 (19%)	27 (9%)

Values are number (%). BNP, brain natriuretic peptide; PAP, pulmonary artery pressure; LV, left ventricle; MI, myocardial infarction; RV, right ventricle.

* Groups are not mutually exclusive as patients may have more than one indication for echocardiography or abnormality.

† Nine patients included in the analysis had missing indications.

‡ The other group includes patients with indication of ventricular arrhythmia, tamponade, circulatory shock, and a combination of free-text indications such as suspected endocarditis, or pulmonary embolus.

§ Severe cardiac disease is defined as severe left ventricular or right ventricular dysfunction or cardiac tamponade.

## Patients without pre-existing cardiac disease

**Figure 4:**
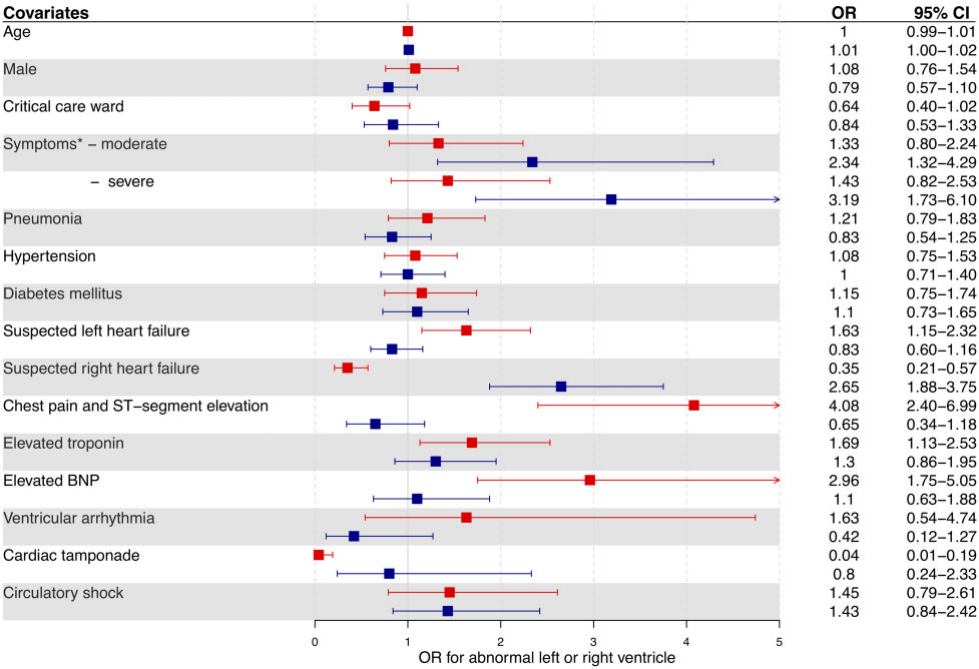
Predictors of an abnormal left (red) and right (blue) ventricle on echocardiography in patients with COVID-19 without pre-existing cardiac disease. Two multivariable logistic regression models examined the associations of clinical covariates with abnormal left ventricular or abnormal right ventricular findings on echocardiography. Categorical covariate data comprised only those answers that were pre-defined in survey questions and were selected *a priori* based on clinical relevance. *Those with mild symptoms were the referent group for symptom severity. BNP, brain type natriuretic peptide.

After excluding 315 patients with pre-existing ischaemic heart disease, heart failure, or valvular heart disease, 901 patients were identified [60 (50–69) years, 68% male] (Supplementary material online, *Table S2*). These patients were more likely to have a normal echocardiogram (54%, 488/901) than those with pre-existing heart disease (19%, 61/315; *P* < 0.001). Suspected left ventricular failure was the most common indication for scanning (35%), followed by suspected right heart failure (23%) and elevated cardiac biomarker concentration (23%). A quarter of patients had an abnormal left ventricle and a third had an abnormal right ventricle. On both univariable and multivariable analysis, the covariates and indications for echocardiography that predicted an abnormal left and right ventricle differed (*Figure 4*; Supplementary material online, *Tables S3* and *S4*). The independent predictors of an abnormal left ventricle were suspected left heart failure [odds ratio (OR) 1.63, 95% confidence interval (CI) 1.15–2.32], chest pain with ST-segment elevation (OR 4.08, 95% CI 2.40–6.99), troponin elevation (OR 1.69, 95% CI 1.13–2.53), and BNP elevation (OR 2.96, 95% CI 1.75–5.05). In contrast, the independent predictors of an abnormal right ventricle were suspected right heart failure (OR 2.65, 95% CI 1.88–3.75) and moderate (OR 2.34, 95% CI 1.32–4.29) or severe COVID-19 symptoms (OR 3.19, 95% CI 1.73–6.10). Overall, 1 in 8 of these patients without pre-existing cardiac disease (13%) had severe cardiac disease identified on echocardiography.

## Changes in management

In 405 (33%) patients, an immediate change in management due to the echocardiogram was reported (*Table 1*). In the remaining 811 (67%) patients, no change in management was reported in 675 (56%), or it was not clear to the echocardiographer whether there had been a change in management in 136 (11%), as the individuals performing the scan were not directly involved in guiding the patients’ care. An immediate change in management occurred more often in those patients with an abnormal compared with a normal echocardiogram (45% vs. 20%, *P* < 0.001, *Figure 3*) and similarly in those with severe disease compared with those with no severe disease identified on echocardiogram (59% vs. 29%, *P* < 0.001; Supplementary material online, *Table S1*). Specific changes in management reported in the free-text comments were collated into four groups. In patients in whom a change in management was reported, the echocardiogram led to changes in disease-specific therapy in 42% (171/405), such as initiating therapy for heart failure, acute coronary syndrome, tamponade, or pulmonary embolism, and commencing antimicrobial therapy for endocarditis. The echocardiogram also facilitated decisions regarding changes in the level of patient care in 8% (32/405), and guided titration of haemodynamic support in 13% (51/405). In the remaining 37% (151/405) where management changed, the change was not described. Changes in management were reported in a higher proportion of patients with pre-existing cardiac disease compared with those without (38% vs. 32%, *P* = 0.005), and in those with elevated cardiac biomarkers compared with the remaining population (39% vs. 31%, *P* < 0.001).

## Discussion

We report findings from the first international survey of echocardiography in patients with confirmed or suspected COVID-19. Data from 1216 patients scanned in 69 countries across six continents demonstrated left or right ventricular abnormalities in half of all patients with COVID-19 undergoing echocardiography, and that these abnormalities were severe in 1 in 7 patients. The majority had non-specific patterns of ventricular dysfunction, although new myocardial infarction, myocarditis, and takotsubo cardiomyopathy were observed in a minority of patients. Echocardiography was reported to directly change patient management in a third of cases including alterations to disease-specific management, haemodynamic support, and the level of care received by the patients.

The simple online format of this survey allowed rapid capture of the echocardiographic findings from a large number of patients with COVID-19 during the pandemic’s peak. This was facilitated by our ability to disseminate and to publicize the survey via social media and through an established global network of imaging specialists. This format allowed us to keep pace with the rapid spread of COVID-19 around the world. Most scans were performed in the current epicentres of the outbreak: the UK, Italy, Spain, France, and the USA. While undoubtedly a global survey, our data remain representative of the current geographical distribution of the virus.

Whilst our previous understanding of how COVID-19 affects the heart was limited to case reports and case series,^7–9^ consistent epidemiological data have demonstrated that patients with established cardiovascular disease, risk factors, or elevated cardiac biomarkers have an increased susceptibility to infection and an increased risk of severe disease and death.^3–6^ Severe cardiac disease was observed in 1 in 7 patients across the whole cohort and in 1 in 8 patients without pre-existing cardiac disease. This proportion rose to 1 in 5 when the indication for imaging included raised cardiac biomarkers. The proportion of abnormal echocardiograms and those demonstrating severe cardiac disease were similar after excluding patients with previously established cardiac disease (heart failure, valve disease, or ischaemic heart disease), suggesting that in this population the cardiac abnormalities relate to COVID-19 infection.

The pattern of cardiac injury observed in our survey appears to be consistent with the cardiovascular involvement observed in patients with other severe viral respiratory infections.^16–19^ Right ventricular abnormalities were observed in a quarter of patients and were more common in patients with more severe symptoms of COVID-19. These are likely to reflect severe respiratory disease, including the viral pneumonia itself, as well as both clinical and subclinical pulmonary thrombo-embolism.^20^ Left ventricular abnormalities were present in a third of patients and were predominantly non-specific in nature. Further research is required to define the mechanism of this dysfunction as only occasionally were echocardiographic patterns consistent with myocardial infarction, myocarditis, or takotsubo cardiomyopathy. The latter conditions are often difficult to recognize during an isolated echocardiogram, particularly when performed in a critical care setting, and, as such, their true prevalence may have been underestimated.

In a third of patients who underwent echocardiography on clinical indication, imaging was reported to result in an immediate change in patient management. This included changes in disease-specific therapies, such as pericardiocentesis or therapy for heart failure, pulmonary embolism, or acute coronary syndromes. It also contributed to decisions regarding the level of patient care, such as the admission of patients to critical care, and the need for titration of haemodynamic support. In practice, this proportion may have been underestimated as echocardiographers may not have fully appreciated the consequences of their scan at the time of imaging. In addition, a majority of patients had echocardiography performed in an intensive care unit. In this setting, optimization of management may have been previously instituted or changes in management limited by severe respiratory or haemodynamic compromise. Few previous studies have reported the impact of echocardiography on changes in management, and none has been performed in a critical care setting.^21^ To put our findings into context, Bethge *et al.* report in an outpatient setting that whilst 22% of patients had abnormal findings, management changed in only 3% of patients.^22^ Finally, we suggest that information supporting the continuation of a management strategy may be as clinically relevant as information that leads to the initiation of an alternative strategy.

The complex logistics involved in performing echocardiography in patients with COVID-19 and the risk of virus transmission necessitates robust selection of patients for imaging.^23^ Our data do not imply that all patients with COVID-19 require an echocardiogram. Indeed, patients undergoing echocardiography here had clearly defined clinical indications. Our data suggest that cardiac biomarkers may help improve the selection of patients for imaging, with elevated BNP and cardiac troponin concentrations independent predictors of left and right ventricular abnormalities, respectively. Building on this study, there is now a need for future imaging and biomarker studies to systematically investigate the cardiovascular manifestations of COVID-19, and to establish their true prevalence. The CAPACITY-COVID European Registry aims to determine the role of cardiovascular disease in the COVID-19 pandemic through standardized large-scale data collection.^24^ Imaging with echocardiography and cardiovascular magnetic resonance following recovery from COVID-19 will be more readily achievable and will be well placed to define any residual cardiac damage caused by the condition. Similarly, studies investigating whether cardiac biomarkers can better direct clinical imaging and improve patient outcomes would be welcome.

Our study suffers from the usual limitations associated with an observational survey. Whilst by design we sought to conduct a rapid survey capturing key echocardiographic findings during the pandemic’s peak, this limited the amount and granularity of the data we could capture. We are reliant on operator-reported findings, as is common in clinical practice, and acknowledge that definitive assessment and core lab verification of cardiac function with echocardiography in critically ill patients is challenging. A proportion of the data was collected from free text-fields, and as such may be biased and represent an underestimate of these findings or clinical variables. Additionally, this survey is subject to substantial case selection bias. For example, we do not know the prevalence of abnormalities in those who did not undergo scanning. In view of the complex logistics around scanning, echocardiography was probably limited to those with clear clinical indications or those with increased disease severity. Furthermore, the use of echocardiography has probably decreased in the current pandemic due to concerns over viral transmission, and this may further contribute to the selection of patients for scanning. We did not capture patient outcomes, but many of the relevant outcomes have yet to occur. Finally, there were relatively few data from certain countries, including China. As the survey continues, we will seek to better target and gather more information from these countries, with further reports to follow.

In this global survey, cardiac abnormalities were observed in half of all COVID-19 patients undergoing echocardiography. Abnormalities were often unheralded or severe, and imaging changed management in one-third of patients.

## Supplementary material

Supplementary material is available at *European Heart Journal – Cardiovascular Imaging* online.

## Supplementary Material

jeaa178_Supplementary_Data
