# Supplementary material for: Global evaluation of echocardiography in patients with COVID-19
Source: Eur Heart J Cardiovasc Imaging. 2020 Jun 18;21(9):949–58. doi: 10.1093/ehjci/jeaa178 (PMC7337658; doi:10.1093/ehjci/jeaa178)
Supplement: jeaa178_Supplementary_Data [file jeaa178_supplementary_data.doc]

**SUPPLEMENTARY DATA**

**Global Evaluation of Echocardiography in Patients with COVID-19**

Marc R. Dweck, M.D.^1^ Anda Bularga, M.D.,^1^ Rebecca Hahn, M.D.,^2^ Rong Bing, MBBS, ^1^

Kuan Ken Lee, M.D., ^1^ Andrew R. Chapman, M.D., ^1^ Audrey White,^1^

Giovanni Di Salvo, M.D., ^3^ Elif Sade, M.D., ^4^ Keith Pearce,^5^ David E. Newby, M.D.,^1^

Bogdan A. Popescu, M.D., ^6^ Erwan Donal, M.D., ^7^ Bernard Cosyns, M.D., ^8^

Thor Edvardsen, M.D.,^9,10^ Nicholas L Mills, M.D.,^1,11^* Kristina Haugaa, M.D. ^9,10^*

^1^ Centre for Cardiovascular Science, University of Edinburgh, United Kingdom

^2^ New York-Presbyterian Hospital/Columbia University Irving Medical Center, New York, USA

^3^ University Hospital Padua, Paediatric Cardiology, Padua, Italy

^4^ Department of Cardiology, University of Baskent, Ankara, Turkey

^5^ University Hospital South Manchester, Cardiology, Wythenshawe, Manchester, United Kingdom

^6^ Department of Cardiology, University of Medicine and Pharmacy 'Carol Davila'-Euroecolab, Emergency Institute for Cardiovascular Diseases 'Prof. Dr. C. C. Iliescu', Sos. Fundeni 258, 022328, Bucharest, Romania

^7^ University of Rennes, CHU Rennes, Inserm, LTSI-UMR 1099, Rennes, France

^8^ Centrum voor Hart en Vaatziekten (CHVZ), Universitair Ziekenhuis Brussel, Vrij Universiteit van Brussel, Brussels, Belgium.

^9^ Department of Cardiology, Oslo University Hospital, Rikshospitalet, Oslo, Norway.

^10^ Faculty of Medicine, University of Oslo, Oslo, Norway.

^11^ Usher Institute, University of Edinburgh, United Kingdom

*Contributed equally

**Correspondingg Author:**

Dr Marc Dweck
BHF/University Centre for Cardiovascular Science The University of Edinburgh
Chancellor’s Building
49 Little France Crescent
Edinburgh EH16 4SB
United Kingdom

E-mail: marc.dweck@ed.ac.uk

**SUPPLEMENTARY DATA CONTENTS**

**Supplementary Appendix** contains the questions of the online echocardiography survey and the list of participating countries

**Table S1** shows the characteristics, indications and outcomes of echocardiography in patients without pre-existing cardiac disease

**Table S2** shows the univariable logistic regression predictors of an abnormal left ventricle or right ventricle on echocardiography in COVID-19 patients without pre-existing cardiac disease

**Table S3** shows the characteristics, indications and outcomes of echocardiography in those with and without severe cardiac disease

**Table S4** shows the characteristics, indications and outcomes of echocardiography in those with left and right ventricular abnormalities without pre-existing cardiac disease

**Supplementary Appendix**

**Transthoracic Echocardiography in COVID-19 Survey**

<https://www.surveymonkey.com/r/2FBFFQD>

Please make sure you enter the data after each scan completion in a confirmed or high-probability COVID-19 patient. Thank you for your contribution and do not forget to bookmark this link.

Our objective is to capture this information quickly and efficiently in real time following echocardiographic examinations and to therefore gain a better picture of the cardiac manifestations of COVID-19 and the contribution of these patient mortality and morbidity.

1. Country where scan performed (free text)
2. Where in the hospital did you perform the TTE scan? (single answer)
   1. Intensive care unit
   2. High dependency ward
   3. Accident and Emergency Unit
   4. Cath lab
   5. Corona virus ward
   6. Cardiology ward
   7. Out-patient department
   8. Other hospital ward (please specify)
3. Patient Age (enter number)
4. Patient gender
   1. Male
   2. Female
5. Patient condition (select all that apply)
   1. Known COVID-19 positive
   2. High probability of COVID-19
   3. Evidence of pneumonia
6. Severity of symptoms (single answer)
   - 1. Mild
     2. Moderate
     3. Severe
7. Cardiovascular co-morbidities (select all that apply)
   1. Known heart failure
   2. Known ischemic heart disease
   3. Known valve disease
   4. Hypertension
   5. Diabetes Mellitus
   6. Other please specify
8. Indication for scan (select all that apply)
   1. Suspected left sided heart failure
   2. Suspected right heart failure
   3. Chest pain and ST elevation on ECG (myocardial infarction vs myocarditis)
   4. High troponin level
   5. High BNP level
   6. Ventricular arrythmias
   7. Shock
   8. Tamponade
   9. Other please specify
9. Left ventricular function (select all that apply)
   1. Normal
   2. Mildly impaired (ejection fraction 55-45%)
   3. Moderately impaired (ejection fraction 35-45%)
   4. Severely impaired (ejection fraction <35%)
   5. Dilated left ventricle
   6. Evidence of new myocardial infarction
   7. Evidence of myocarditis
   8. Evidence of likely tako-tsubo cardiomyopathy
   9. Tamponade
10. Right ventricular function (select all that apply)
    1. Normal
    2. Mild or moderate RV impairment
    3. Severe RV impairment
    4. RV dilatation
    5. D-shaped left ventricle
    6. Pulmonary hypertension
11. Did your echo scan change management?
    1. Yes or no
    2. If so how? Free text

**Participation in the Survey by Country**

*Number of scans contributed in brackets*

Albania (1)

Algeria (3)

Argentina (3)

Armenia (1)

Australia (4)

Austria (1)

Bangladesh (2)

Belgium (16)

Bosnia and Herzegovina (2)

Brazil (22)

Bulgaria (6)

Canada (4)

Chile (2)

Columbia (6)

Cyprus (6)

Czech Republic (1)

Denmark (2)

Ecuador (4)

Egypt (6)

France (102)

Germany (10)

Greece (14)

Hong Kong (2)

India (6)

Indonesia (3)

Iran (5)

Iraq (1)

Ireland (17)

Israel (1)

Italy (69)

Kenya (1)

Kosovo (1)

Lithuania (1)

Malta (1)

Mexico (10)

Morocco (1)

Myanmar (1)

The Netherlands (11)

Nicaragua (1)

Nigeria (1)

North Macedonia (1)

Norway (7)

Oman (1)

Pakistan (6)

Peru (6)

Philippines (2)

Poland (7)

Portugal (17)

Qatar (1)

Romania (11)

Russia (3)

Saudi Arabia (5)

Serbia (8)

Singapore (1)

Slovenia (1)

Spain (97)

Sweden (17)

Switzerland (28)

Syria (1)

Taiwan (1)

Tanzania (1)

Thailand (2)

Turkey (11)

Ukraine (3)

United Arab Emirates (4)

United Kingdom (513)

United States of America (101)

Vietnam (2)

(Missing values (5))

**Table S1:** Patient characteristics and indications for echocardiography stratified according to whether the echocardiogram showed evidence of severe cardiac disease ***

|  | **Overall**  *(n=1,216)* | **Severe disease**  *(n=182)* | **No severe disease**  *(n=1,034)* | **P-value** † |
| --- | --- | --- | --- | --- |
| ***Age*** | 62 [52, 71] | 63 (50, 74) | 62 [52, 71] | 0.700 |
| ***Sex*** |  |  |  | 0.200 |
| Female | 365 (30%) | 62 (34%) | 303 (29%) |  |
| Male | 844 (70%) | 118 (66%) | 726 (71%) |  |
| **L*ocation* of scan** |  |  |  | 0.500 |
| Critical care | 726 (60%) | 104 (57%) | 622 (60%) |  |
| Non-critical care | 486 (40%) | 78 (43%) | 408 (40%) |  |
| ***COVID-19 status*** |  |  |  | 0.600 |
| Confirmed COVID-19 | 813 (73%) | 119 (71%) | 694 (74%) |  |
| High-probability of COVID-19 | 298 (27%) | 48 (29%) | 250 (26%) |  |
| Evidence of pneumonia | 232 (19%) | 32 (18%) | 200 (19%) | 0.600 |
| ***Symptom severity*** |  |  |  | 0.001 |
| Mild | 215 (18%) | 16 (9%) | 199 (20%) |  |
| Moderate | 327 (28%) | 50 (28%) | 277 (28%) |  |
| Severe | 625 (54%) | 112 (63%) | 513 (52%) |  |
| ***Co-morbidities*** |  |  |  |  |
| Hypertension | 445 (37%) | 63 (35%) | 382 (37%) | 0.600 |
| Diabetes mellitus | 233 (19%) | 37 (20%) | 196 (19%) | 0.700 |
| Ischaemic heart disease | 167 (14%) | 30 (16%) | 137 (13%) | 0.300 |
| Heart failure | 113 (9%) | 41 (23%) | 72 (7%) | <0.001 |
| Valvular heart disease | 80 (7%) | 12 (7%) | 68 (7%) | >0.900 |
| ***Indication*** |  |  |  |  |
| Suspected left heart failure | 491 (40%) | 81 (45%) | 410 (40%) | 0.300 |
| Suspected right heart failure | 243 (20%) | 40 (22%) | 203 (20%) | 0.400 |
| Chest pain and ST-elevation | 107 (9%) | 11 (6%) | 96 (9%) | 0.200 |
| Elevated cardiac biomarkers | 314 (26%) | 62 (34%) | 252 (24%) | 0.008 |
| Troponin | 239 (20%) | 44 (24%) | 195 (19%) | 0.140 |
| BNP | 129 (11%) | 33 (18%) | 96 (9%) | 0.002 |
| Ventricular arrhythmia | 38 (3%) | 10 (6%) | 28 (3%) | 0.086 |
| Cardiac tamponade | 20 (2%) | 10 (6%) | 10 (1%) | <0.001 |
| Circulatory shock | 95 (8%) | 26 (14%) | 69 (7%) | 0.002 |
| ***Change* in *management*** |  |  |  | <0.001 |
| Yes | 405 (33%) | 108 (59%) | 297 (29%) |  |
| No | 675 (56%) | 56 (31%) | 619 (60%) |  |
| Not known | 136 (11%) | 18 (10%) | 118 (11%) |  |
| ***Management group*** |  |  |  |  |
| Disease-specific therapy | 171 (14%) | 53 (29%) | 118 (11%) |  |
| Level of care | 32 (3%) | 10 (6%) | 22 (2%) |  |
| Haemodynamic support | 51 (4%) | 14 (7%) | 37 (4%) |  |
| Other | 151 (12%) | 31 (17%) | 120 (12%) |  |
| Median [interquartile interval], number (%). Abbreviations: BNP = B-type natriuretic peptide; COVID-19 = coronavirus disease 2019. * Severe cardiac disease = severe left or right ventricular dysfunction or tamponade. † Between group comparisons are Chi-square test or independent samples t-tests*.* | | | | |

**Table S2: Patients without pre-existing cardiac disease**

Patient characteristics and indications for echocardiography in patients without pre-existing heart disease stratified according to whether the echocardiogram was normal or abnormal

|  | **Overall**  *(n=901)* | **Abnormal scan**  *(n=413)* | **Normal scan**  *(n=488)* | **P-value*** |
| --- | --- | --- | --- | --- |
| ***Age*** | 60 [50, 69] | 61 [50, 71] | 59 [50, 67] | 0.050 |
| ***Sex*** |  |  |  | 0.600 |
| Female | 286 (32%) | 135 (33%) | 151 (31%) |  |
| Male | 610 (68%) | 275 (67%) | 335 (69%) |  |
| **L*ocation* of scan** |  |  |  | >0.900 |
| Critical care | 580 (65%) | 267 (65%) | 313 (64%) |  |
| Non-critical care | 318 (35%) | 145 (35%) | 173 (36%) |  |
| ***COVID-19 status*** |  |  |  | 0.027 |
| Confirmed COVID-19 | 647 (77%) | 283 (74%) | 364 (80%) |  |
| High-probability of COVID-19 | 192 (23%) | 102 (26%) | 90 (20%) |  |
| Evidence of pneumonia | 156 (17%) | 72 (17%) | 84 (17%) | >0.900 |
| ***Symptom severity*** |  |  |  | 0.008 |
| Mild | 146 (17%) | 51 (13%) | 95 (21%) |  |
| Moderate | 197 (23%) | 93 (23%) | 104 (23%) |  |
| Severe | 520 (60%) | 257 (64%) | 263 (57%) |  |
| ***Co-morbidities*** |  |  |  |  |
| Hypertension | 342 (38%) | 171 (41%) | 171 (35%) | 0.058 |
| Diabetes mellitus | 166 (18%) | 83 (20%) | 83 (17%) | 0.300 |
| ***Indication*** |  |  |  |  |
| Suspected left heart failure | 312 (35%) | 144 (35%) | 168 (34%) | >0.900 |
| Suspected right heart failure | 203 (23%) | 109 (26%) | 94 (19%) | 0.036 |
| Chest pain and ST-elevation | 72 (8%) | 44 (11%) | 28 (6%) | 0.022 |
| Elevated cardiac biomarkers | 206 (23%) | 122 (30%) | 84 (17%) | <0.001 |
| Troponin | 163 (18%) | 95 (23%) | 68 (14%) | 0.001 |
| BNP | 77 (9%) | 55 (13%) | 22 (5%) | <0.001 |
| Ventricular arrhythmia | 16 (2%) | 13 (3%) | 3 (1%) | 0.013 |
| Cardiac tamponade | 18 (2%) | 11 (3%) | 7 (1%) | 0.400 |
| Circulatory shock | 74 (8%) | 47 (11%) | 27 (6%) | 0.005 |
| ***Change* in *management*** |  |  |  | <0.001 |
| Yes | 285 (32%) | 195 (47%) | 90 (18%) |  |
| No | 501 (56%) | 173 (42%) | 328 (67%) |  |
| Not known | 115 (13%) | 45 (11%) | 70 (14%) |  |
| ***Management group*** |  |  |  | <0.001 |
| Disease-specific therapy | 127 (14%) | 94 (23%) | 33 (7%) |  |
| Level of care | 25 (3%) | 14 (3%) | 11 (2%) |  |
| Haemodynamic support | 37 (4%) | 23 (6%) | 14 (3%) |  |
| Other | 96 (11%) | 64 (15%) | 32 (7%) |  |
| Median [interquartile interval], number (%). Abbreviations: BNP = B-type natriuretic peptide; COVID-19 = coronavirus disease 2019. * Between group comparisons are Chi-square test or independent samples t-tests. | | | |  |

**Table S3:** Univariable logistic regression for predictors of an abnormal left and right ventricle in patients with COVID-19 without pre-existing cardiac disease

| **Covariates** | **Odds Ratio for LV abnormality** *[95% CI]* | **Odds Ratio for RV abnormality** *[95% CI]* |
| --- | --- | --- |
| ***Age*** | 1.00 [0.99 – 1.01] | 1.01 [1.00-1.02] |
| ***Sex*** |  |  |
| Male | 1.00 [0.74-1.38] | 0.83 [0.61-1.12] |
| ***Location of scan*** |  |  |
| Critical care | 0.65 [0.48-0.87] | 1.38 [1.02-1.88] |
| ***Symptom severity*** |  |  |
| Moderate | 1.22 [0.77-1.95] | 2.36 [1.37-4.20] |
| Severe | 0.88 [0.59 – 1.33] | 3.34 [2.08-5.62] |
| ***Evidence of pneumonia*** | 1.24 [0.85-1.79] | 0.93 [0.63-1.35] |
| ***Co-morbidities*** |  |  |
| Hypertension | 1.21 [0.90-1.63] | 1.20 [0.89-1.60] |
| Diabetes mellitus | 1.28 [0.88-1.84] | 1.11 [0.77-1.59] |
| ***Indication*** |  |  |
| Suspected left heart failure | 1.11 [0.96-1.28] | 0.86 [0.68-1.02] |
| Suspected right heart failure | 0.71 [0.51-0.93] | 1.16 [1.01-1.36] |
| Chest pain and ST-segment elevation | 1.15 [0.98-1.34] | 0.85 [0.62-1.03] |
| Elevated troponin | 1.15 [1.00-1.35] | 0.95 [0.78-1.11] |
| Elevated BNP | 1.15 [0.99-1.35] | 0.95 [0.77-1.12] |
| Cardiac tamponade | 0.98 [0.80-1.16] | 0.91 [0.69-1.09] |
| Ventricular arrhythmia | 1.03 [0.86-1.21] | 0.89 [0.66-1.07] |
| Circulatory shock | 1.04 [0.88-1.22] | 0.99 [0.82-1.15] |
| Abbreviations: LV = left ventricle; RV = right ventricle; CI = confidence interval. | | |

**Table S4:** Patient characteristics and indications for echocardiography in those without pre-existing cardiac disease and left or right ventricular abnormalities

|  | **LV abnormality**  *(n=250)* | **RV abnormality**  *(n=271)* |
| --- | --- | --- |
| ***Age*** | 62 [49, 71] | 60 [50, 72] |
| ***Sex*** |  |  |
| Female | 79 (32%) | 94 (35%) |
| Male | 169 (68%) | 176 (65%) |
| **L*ocation* of scan** |  |  |
| Critical care | 143 (57%) | 188 (70%) |
| Non-critical care | 107 (43%) | 82 (30%) |
| ***COVID-19 status*** |  |  |
| Confirmed COVID-19 | 158 (68%) | 200 (79%) |
| High-probability of COVID-19 | 74 (32%) | 53 (21%) |
| Evidence of pneumonia | 49 (20%) | 45 (17%) |
| ***Symptom severity*** |  |  |
| Mild | 42 (17%) | 21 (8.0%) |
| Moderate | 65 (27%) | 56 (21%) |
| Severe | 136 (56%) | 187 (71%) |
| ***Co-morbidities*** |  |  |
| Hypertension | 103 (41%) | 111 (41%) |
| Diabetes mellitus | 53 (21%) | 53 (20%) |
| ***Indication*** |  |  |
| Suspected left heart failure | 107 (43%) | 86 (32%) |
| Suspected right heart failure | 30 (12%) | 101 (37%) |
| Chest pain and ST-elevation | 44 (18%) | 17 (6.3%) |
| Elevated cardiac biomarkers | 94 (38%) | 67 (25%) |
| Troponin | 72 (29%) | 54 (20%) |
| BNP | 46 (18%) | 29 (11%) |
| Ventricular arrhythmia | 11 (4%) | 5 (2%) |
| Cardiac tamponade | 5 (2%) | 7 (3%) |
| Circulatory shock | 29 (12%) | 33 (12%) |
| ***Change* in *management*** |  |  |
| Yes | 132 (53%) | 127 (47%) |
| No | 92 (37%) | 113 (42%) |
| Not known | 26 (10%) | 31 (11%) |
| ***Management group*** |  |  |
| Disease-specific therapy | 62 (25%) | 58 (21%) |
| Level of care | 13 (5%) | 8 (3%) |
| Haemodynamic support | 13 (5%) | 21 (8%) |
| Other | 44 (18%) | 40 (15%) |
| Median [interquartile interval], number (%). Abbreviations: BNP = B-type natriuretic peptide; COVID-19 = coronavirus disease 2019; LV = left ventricle; RV = right ventricle. | | |
